# Supplementary material for: Fluid shear stress-dependent modulation of the basal endothelial glycocalyx
Source: PLoS One. 2026 Jan 23;21(1):e0339318. doi: 10.1371/journal.pone.0339318 (PMC12829966; doi:10.1371/journal.pone.0339318)
Supplement: S1 Table — The significance level (Sig), P-values (P), and degree of freedom (DF) are reported for each individual comparison generated within the Anova results presented in Figure 1 – Figure 5 (A – E). The overall Anova p-value (P) along with the sum of the treatment and residual degrees of freedom (F) for each glycocalyx (GCX) metric (coverage, integrated intensity, and thickness) are reported. Significance level correspond to the following ranges: (ns: p = > 0.005), (*: p < 0.05), (**: p < 0.01), (***: p < 0.001). Naming samples includes the shear stress exposure time in hours (H) and rate in dynes/cm2 (D) therefore the experimental group exposed to 10 dynes/cm2 of fluid shear stress for 0.5 hours would be labeled as 0.5H10D. Non-permeabilized (NP), permeabilized (P). (DOCX) [file pone.0339318.s003.docx]

| 1. **Fig. 1:** Comparison of permeabilization effects on apical glycocalyx (GCX) metrics | | | | | | | | | | | |
| --- | --- | --- | --- | --- | --- | --- | --- | --- | --- | --- | --- |
|  | **Coverage** | | |  | **Integrated Intensity** | | |  | **Thickness** | | |
| **Comparison** | **F/Sig** | **P** | **DF** |  | **F/Sig** | **P** | **DF** |  | **F/Sig** | **P** | **DF** |
| Overall ANOVA | 3.818 | 0.0096 | 32 |  | 3.219 | 0.0208 | 32 |  | 2.333 | 0.0414 | 3.604 |
| Static NP vs Static P | ns | 0.9778 | 0.3638 |  | ns | >0.9999 | 0.01032 |  | ns | 0.9720 | 299.4 |
| 0.5H10D NP vs 0.5H10D P | ns | 0.3128 | 1.617 |  | ns | 0.6497 | 1.068 |  | ns | 0.7784 | 90.77 |
| 12H10D NP vs 0.5H10D P | ns | 0.1208 | 2.135 |  | ns | 0.1323 | 2.090 |  | ns | 0.4261 | 71.24 |
| 1. **Fig. 2:** Apical and basal expression after exposure to 10 dyne/cm^2^ | | | | | | | | | | | |
| Overall ANOVA | 28.49 | <0.0001 | 31 |  | 61.40 | <0.0001 | 31 |  | 60.47 | <0.0001 | 69.69 |
| Static A vs Static B | **** | <0.0001 | 26 |  | **** | <0.0001 | 26 |  | **** | <0.0001 | 531.8 |
| 0.5H A vs 0.5H B | **** | <0.0001 | 26 |  | **** | <0.0001 | 26 |  | **** | <0.0001 | 121.7 |
| 12H A vs 12H B | * | 0.0469 | 26 |  | **** | <0.0001 | 26 |  | **** | <0.0001 | 83.55 |
| Static A vs 0.5H A | * | 0.0205 | 26 |  | ns | 0.1714 | 26 |  | ns | 0.8565 | 126.7 |
| Static A vs 12H A | ns | 0.0593 | 26 |  | ns | 0.5794 | 26 |  | ns | 0.9102 | 76.29 |
| 0.5H A vs 12H A | **** | <0.0001 | 26 |  | ** | 0.0038 | 26 |  | ns | >0.9999 | 92.97 |
| Static B vs 0.5H B | ns | 0.0832 | 26 |  | **** | <0.0001 | 26 |  | * | 0.0138 | 96.65 |
| Static B vs 12H B | ns | 0.9997 | 26 |  | ns | >0.9999 | 26 |  | **** | <0.0001 | 66.16 |
| 0.5H B vs 12H B | ns | 0.3134 | 26 |  | **** | <0.0001 | 26 |  | **** | <0.0001 | 98.99 |
| 1. **Fig. 3:** Apical and basal expression after exposure to 0.5 dyne/cm^2^ | | | | | | | | | | | |
| Overall ANOVA | 33.49 | <0.0001 | 31 |  | 58.57 | <0.0001 | 31 |  | 17.59 | <0.0001 | 17.22 |
| Static A vs Static B | **** | <0.0001 | 26 |  | **** | <0.0001 | 26 |  | **** | <0.0001 | 350.8 |
| 0.5H A vs 0.5H B | **** | <0.0001 | 26 |  | **** | <0.0001 | 26 |  | *** | 0.0009 | 74.45 |
| 12H A vs 12H B | * | 0.0237 | 26 |  | ** | 0.0214 | 26 |  | **** | <0.0001 | 181.3 |
| Static A vs 0.5H A | ns | 0.3362 | 26 |  | ns | 0.9954 | 26 |  | ns | 0.9512 | 62.92 |
| Static A vs 12H A | * | 0.0282 | 26 |  | ns | 0.0919 | 26 |  | ns | 0.9965 | 206.1 |
| 0.5H A vs 12H A | ns | 0.9513 | 26 |  | ns | 0.4717 | 26 |  | ns | 0.7136 | 76.29 |
| Static B vs 0.5H B | ns | >0.9999 | 26 |  | ns | 0.9431 | 26 |  | ns | 0.9976 | 59.02 |
| Static B vs 12H B | **** | <0.0001 | 26 |  | **** | <0.0001 | 26 |  | * | 0.0106 | 200.7 |
| 0.5H B vs 12H B | **** | <0.0001 | 26 |  | **** | <0.0001 | 26 |  | ns | 0.6123 | 72.81 |
| 1. **Fig. 4:** Apical and basal expression after exposure to 30 dyne/cm^2^ | | | | | | | | | | | |
| Overall ANOVA | 22.05 | <0.0001 | 37 |  | 72.17 | <0.0001 | 37 |  | 38.06 | <0.0001 | 47.61 |
| Static A vs Static B | **** | <0.0001 | 32 |  | **** | <0.0001 | 32 |  | ** | 0.0037 | 179.1 |
| 0.5H A vs 0.5H B | ** | 0.0098 | 32 |  | ** | 0.0014 | 32 |  | **** | <0.0001 | 214.1 |
| 12H A vs 12H B | * | 0.0112 | 32 |  | *** | 0.0001 | 32 |  | **** | <0.0001 | 203.1 |
| Static A vs 0.5H A | * | 0.0400 | 32 |  | * | 0.0366 | 32 |  | ns | 0.9983 | 182.8 |
| Static A vs 12H A | ns | 0.9998 | 32 |  | ns | 0.8947 | 32 |  | ** | 0.0035 | 147.8 |
| 0.5H A vs 12H A | ns | 0.1066 | 32 |  | ns | 0.4212 | 32 |  | ** | 0.0042 | 198.9 |
| Static B vs 0.5H B | **** | <0.0001 | 32 |  | **** | <0.0001 | 32 |  | ns | 0.1922 | 203.6 |
| Static B vs 12H B | ** | 0.0036 | 32 |  | **** | <0.0001 | 32 |  | * | 0.0422 | 209.9 |
| 0.5H B vs 12H B | ns | 0.2155 | 32 |  | ns | 0.1858 | 32 |  | ns | >0.9999 | 222.1 |
| 1. **Fig. 5:** Comparison across shear rates | | | | | | | | | | | |
| 0.5H10D A vs 0.5H0.5D A | ns | 0.6464 | 42 |  | ns | 0.1648 | 42 |  | ns | 0.9872 | 1300 |
| 0.5H10D A vs 0.5H30D A | ns | 0.7621 | 42 |  | ns | 0.1484 | 42 |  | ns | 0.8696 | 1300 |
| 0.5H0.5D A vs 0.5H30D A | ns | 0.9712 | 42 |  | ns | 0.9996 | 42 |  | ns | 0.9180 | 1300 |
| 0.5H10D B vs 0.5H0.5D B | ** | 0.0092 | 42 |  | **** | <0.0001 | 42 |  | ns | 0.2821 | 1300 |
| 0.5H10D B vs 0.5H30D B | **** | <0.0001 | 42 |  | **** | <0.0001 | 42 |  | ns | 0.3950 | 1300 |
| 0.5H0.5D B vs 0.5H30D B | *** | 0.0009 | 42 |  | ns | 0.0583 | 42 |  | ns | 0.9035 | 1300 |
| 12H10D A vs 12H0.5D A | ns | 0.1047 | 42 |  | **** | <0.0001 | 42 |  | ns | 0.4024 | 1300 |
| 12H10D A vs 12H30D A | ns | 0.8146 | 42 |  | ns | 0.0519 | 42 |  | *** | 0.0003 | 1300 |
| 12H0.5D A vs 12H30D A | * | 0.0174 | 42 |  | ** | 0.0057 | 42 |  | ns | 0.1967 | 1300 |
| 12H10D B vs 12H0.5D B | **** | <0.001 | 42 |  | **** | <0.0001 | 42 |  | **** | <0.0001 | 1300 |
| 12H10D B vs 12H30D B | ** | 0.0084 | 42 |  | ns | 0.1450 | 42 |  | ns | 0.9841 | 1300 |
| 12H0.5D B vs 12H30D B | *** | 0.0007 | 42 |  | **** | <0.0001 | 42 |  | **** | <0.0001 | 1300 |
