# Supplementary material for: Fluid shear stress-dependent modulation of the basal endothelial glycocalyx
Source: PLoS One. 2026 Jan 23;21(1):e0339318. doi: 10.1371/journal.pone.0339318 (PMC12829966; doi:10.1371/journal.pone.0339318)
Supplement: Attachment — Submitted filename: ResponseLetter.docx [file pone.0339318.s005.docx]

**Title:** Fluid shear stress-dependent modulation of the basal endothelial glycocalyx

**Journal:** PLOS One

**Submission ID:** PONE-D-25-37102

**Reviewer #1:**

1. Cover Sheet Inconsistencies:

**1.1.** The funding disclosure states no support was received, yet institutional funding is mentioned in the manuscript. This should be corrected to ensure proper acknowledgment.
**1.2.** The data availability statement includes a minor typographical error: “analyzed the presented” likely should read “analyzed and presented.”

**Response to Reviewer**

We thank the reviewer for the helpful comments. We apologize for the inconsistencies in the initial submission. Upon review, it appears that the funding disclosure was inadvertently placed in the competing interest’s section.

**Changes made to manuscript:**

“All image data that supports the results of this study, as well as all novel ImageJ macro-functions and CellProfiler pipeline generated for the analysis of the image data collected, have been stored in the DRYAD database which can be accessed at DIO : (available after publication). Both authors had access to all data included in the study presented.” – page 17 lines 566-569

“Funding disclosure: This work was funded by startup funding awarded to Prof. Solomon A. Mensah by Worcester Polytechnic Institute.

Competing interest statement: The authors declare that no competing interests exist.” - page 18 lines 543-546

***2*.** Introduction Clarity:

**2.1.** The mention of turbulence is vague. Does turbulent flow occur in the body, and under what conditions? Clarifying this would strengthen the physiological relevance.

**Response to Reviewer**

We appreciate the reviewer’s insightful comment. We do not believe that true turbulent flow occurs under normal physiological conditions within the human vasculature. Rather, disturbed flow, characterized by regions of flow separation, recirculation, and oscillation, has been well documented at sites of vascular bifurcations and curvatures, where local Reynolds numbers remain below those required for full turbulence. To address this, we have clarified the physiological relevance of our experimental flow conditions. The following excerpt has been added to the Introduction to highlight how disturbed flow contributes to endothelial dysfunction and vascular disease susceptibility:

We have also added the second paragraph to the discussion to more concretely connect our experimental hypothesis to both our experimental design and physiological relevance. Hopefully clarifying uncertainties around the novelty and relevance of the data collected and presented.

**Changes made to manuscript:**

“Vessel curvature and bifurcations create regional variations in FSS due to the complex and multidirectional flow patterns that develop in these areas^1-3^. Endothelial permeability and disease susceptibility have been shown to correlate with fluctuations in the time-averaged FSS over a pulsatile cardiac cycle, as well as with the oscillatory nature of the local shear environment ^2,4,5^. The apical GCX which directly interfaces with circulating blood, has been extensively investigated under these varying shear conditions, revealing numerous mechanistic links between FSS and apical GCX-mediated endothelial mechanotransduction^6,7^..” – page 2, lines 47 - 56

“The in vitro flow conditions used in this study were designed to mimic the flow profiles typical of the vasculature. By examining EC and GCX responses under these shear environments, our findings provide insights into how local hemodynamic forces drive endothelial GCX response and gradients across the endothelium.” – page 12 lines 377-380

**2.2.** The hypothesis is not explicitly stated. A clear hypothesis would help frame the study and guide interpretation of the results.

**Response to Reviewer**

We thank the reviewer for this helpful suggestion. A clarifying statement has been added to the Introduction to better frame the study hypothesis. Together with the revisions in the discussion, these additions more clearly highlight the study’s rationale, hypothesis, and translational relevance:

**Changes made to manuscript:**

“We hypothesize that FSS elicits coordinated responses in both the apical and basal GCX through apical GCX and cytoskeletal mediated mechanotransduction^8,9^. Furthermore we hypothesize the response of the apical and basal GCX will also be dependent upon the magnitude of FSS applied^10^. ” – page 3 lines 82-85

**3.** Methods Presentation:
**3.1.** Supplemental Figure 3 could be in the main body of the paper

**Response to Reviewer**

We thank the reviewer for this helpful observation. We agree that the figure provides important context for the main methods presented in the paper. Accordingly, we have moved the Supplementary Figure 3 and its caption to the main body of the manuscript as Figure 6 in the methods section (page 16, lines 474-492).

**4.** Results Presentation:
**4.1.** The emphasis on permeabilization vs. non-permeabilization is confusing given that glycocalyx is extracellular. The rationale for this distinction should be clarified.
**4.2.** Figures 2 to 5 do not indicate whether cells were permeabilized.

**Response to Reviewer**

We thank the reviewer for this insightful comment. We agree that the emphasis on permeabilization versus non-permeabilization requires further clarification given that the GCX is primarily an extracellular structure. Traditionally, permeabilization has not been incorporated into immunofluorescent staining protocols for the GCX, as it was largely assumed to be confined to the apical surface of endothelial cells (Reitsma, 2007).

However, in the context of the basal GCX, permeabilization becomes necessary because the antibodies used must access the endothelial basement membrane to tag basal GCX components. To address this, we performed a dose-response study comparing two detergents—Triton X-100 and sucrose/saponin, to determine which permeabilization conditions would best preserve GCX integrity. Supplementary Figure 2 demonstrated that sucrose/saponin treatment was gentler on the GCX, with no significant differences observed between apical GCX metrics compared between treated and non-treated samples post flow samples (Figure 2). It was also clarified in text that Figures 2 – 5 show results for permeabilized HLMVECs as we are probing apical and basal GCX in these comparisons. Accordingly, we revised the relevant section of the manuscript as follows:

**Changes made to manuscript:**

“Most reports of the endothelial GCX have not included permeabilization during immunofluorescent staining, based on the long-standing assumption that the GCX exists solely on the luminal surface^11^. As shown in prior studies, staining with WGA in the absence of permeabilization labels only the apical GCX of confluent endothelial monolayers^12-14^. In contrast, when EC are tested with sucrose/saponin, the basal GCX can be successfully visualized^15^. 2% Sucrose and 0.1% saponin were used for membrane stabilization and permeabilization, respectively, over Triton-X100, a common detergent, as initial dose-dependent studies for basal GCX visualization displayed membrane discontinuities post permeabilization. The brightfield images revealed disruptions in the cell membrane, which were further confirmed by GCX staining in the orthogonal views (Supplementary Fig 2). These observations validate the membrane disruptions that occurred following permeabilization using Triton-X100. When comparing apical GCX signal intensity, quantified as the GCX signal above the z-stack plane corresponding to the centroid of the nuclei, between non-permeabilized HLMVECs and those permeabilized with 0.1% saponin, no significant differences were observed among HLMVECs exposed to FSS for the same duration (Fig. 1G–I). These results indicate that sucrose/saponin treatment preserves GCX integrity without significantly altering the apical signal (Fig 1). Subsequent data are grouped into apical and basal GCX signals, obtained from HLMVECs that were permeabilized after flow exposure and fixation. The collected z-stacks were separated using the described method to independently quantify apical and basal GCX metrics (Fig 6). ” – page 4-5 lines 116-134

**4.3.** In Figure 1, statistical comparisons across time points are missing. Given the observed decrease at 30 min and recovery at 12 hr, statistical analysis would strengthen the conclusions.

**Response to Reviewer**

We thank the reviewer for this helpful comment. As clarified in Figure 1 and discussed further in Response 4.2, the only comparisons presented in this figure are between the apical GCX signal of permeabilized and non-permeabilized HLMVECs following static culture, 30 minutes, and 12 hours of exposure to 10 dynes/cm² of FSS. The three non-significant comparisons shown represent the only analyses performed for this dataset as the major conclusion drawn from Figure 2 is that the apical GCX signal is preserved when HLMVECs are permeabilized post flow exposure and post fix to allow tagging of the apical and basal GCX.

The temporal FSS responses of both apical and basal GCX metrics, as well as comparisons between apical and basal GCX for each FSS magnitude, are presented in Figures 2–4. Figure 5 further illustrates the relationship between FSS magnitude and the temporal response of apical and basal GCX metrics independently. Figure legends and corresponding sections in the Results have been updated to clarify these distinctions. Figure legends and results have been updated to clarify.

**Changes made to manuscript:**

“Most reports of the endothelial GCX have not included permeabilization during immunofluorescent staining, based on the long-standing assumption that the GCX exists solely on the luminal surface^11^. As shown in prior studies, staining with WGA in the absence of permeabilization labels only the apical GCX of confluent endothelial monolayers^12-14^. In contrast, when EC are tested with sucrose/saponin, the basal GCX can be successfully visualized^15^. 2% Sucrose and 0.1% saponin were used for membrane stabilization and permeabilization, respectively, over Triton-X100, a common detergent, as initial dose-dependent studies for basal GCX visualization displayed membrane discontinuities post permeabilization. The brightfield images revealed disruptions in the cell membrane, which were further confirmed by GCX staining in the orthogonal views (Supplementary Fig 2). These observations validate the membrane disruptions that occurred following permeabilization using Triton-X100. When comparing apical GCX signal intensity, quantified as the GCX signal above the z-stack plane corresponding to the centroid of the nuclei, between non-permeabilized HLMVECs and those permeabilized with 0.1% saponin, no significant differences were observed among HLMVECs exposed to FSS for the same duration (Fig. 1G–I). These results indicate that sucrose/saponin treatment preserves GCX integrity without significantly altering the apical signal (Fig 1). Subsequent data are grouped into apical and basal GCX signals, obtained from HLMVECs that were permeabilized after flow exposure and fixation. The collected z-stacks were separated using the described method to independently quantify apical and basal GCX metrics (Fig 6).

**Fig 1. Comparison of the apical glycocalyx (GCX) signal of permeabilized and non-permeabilized human lung microvascular endothelial cells (HLMVECs) exposed to 10 dynes/cm² of fluid shear stress revealed no statistically significant differences between HLMVECs of the same FSS exposure time.**

Sum projections (enface view of A-F) and accompanying orthogonal views (directly below enface view, A-F) of the apical GCX (green) and nuclei (blue) in non-permeabilized (A-C) and permeabilized (D-F) HLMVECs after static culture (A,D), 30 minutes (B,E), and 12 hours (C,F) of exposure to 10 dynes/cm^2^ fluid shear stress. No significant differences were found between GCX thickness (µm) (G), coverage (%) (H), and integrated intensity (fold-change (F.C.)) (I) of non-permeabilized and permeabilized HLMVECs.**”** – pages 4-5 lines 116 - 144

**4.4.** Supplemental figure data appear inconsistent with the purple bars in Figure 1, though both are said to represent non-permeabilized cells. This discrepancy should be explained.
**Response to Reviewer**

We thank the reviewer for this thoughtful comment. We recognize that the apparent inconsistency between the supplemental figure data and the purple bars in Figure 1 required clarification. In Figure 1, we specifically compare the apical GCX signal between permeabilized and non-permeabilized HLMVECs to demonstrate that permeabilization does not significantly alter apical GCX metrics. The comparison is based solely on what our image segmentation method designates as apical regions. These analyses confirm that the apical GCX signal remains largely unaffected by permeabilization, while basal GCX signal becomes detectable only after permeabilization. Supplementary Figure 1 displays the results for non-permeabilized HLMVECs not separated into apical and basal projections. To further clarify this distinction, we have added representative images to Supplementary Figure 1, revised the Figure 1 caption, and expanded the results section and Supplementary Figure 1 caption to better describe how these data were processed and interpreted.

**Changes made to manuscript:**

“**Supplementary Fig 1. Effect of physiologically normal (10 dynes/cm^2^) fluid shear stress (FSS) on non-permeabilized human lung microvascular endothelial cells (HLMVECs) glycocalyx (GCX) expression.**

Apical GCX thickness (um)(A), coverage (%)(B), and integrated intensity (fold-change (F.C.)(C) in HLMVECs after static culture, 30 minutes, and 12 hours of exposure to physiologically normal (10 dynes/cm^2^) FSS exposure. WGA staining was performed on non-permeabilized post-fix HLMVECs. Here, no apical / basal image separation was performed, and all slices of the full thickness z-stack are summed to a single sum projection and the metrics presented were quantified to ensure the bulk non-permeabilized expression of the HLMVEC GCX displays FSS dependent expression. GCX thickness was analyzed across the entire Z-stacks YZ orthogonal view of the z-stacks collected.” Page 1, lines 4-13

“HLMVECs were confirmed to produce a robust apical GCX in response to physiologically normal FSS by probing the GCX following methods previously used to visualize and quantify the luminal GCX (Supplementary Fig 1)^11,12,14^. Following exposure to 10 dynes/cm² of FSS for 12 hours, HLMVECs were fixed as described, and their GCX was labeled without permeabilization to ensure that only components naturally accessible to antibodies were tagged during immunostaining. Full thickness z-stacks were not separated using the apical / basal image separate process described, instead entire z-stack collected was sum projected and quantified. Metrics of coverage and integrated intensity were found to significantly increase compared to static conditions (p = 0.0014, p = 0.0135 respectively), and the levels observed after 30 minutes of exposure (p = 0.0002, p = 0.0113 respectively) (Supplementary Fig 1). This indicates a sustained and overall enhancement in GCX expression in response to prolonged shear stress. Measurements of GCX thickness (µm) correspond with the literature ranges reported (Supplementary Fig 1)^12,16,17^. These results align with what has been reported for prominent GCX components in other endothelial cell types, such as HUVECs when using the typically presented method of GCX staining, visualization, and quantification^14,18-21^. It is also important to note that when z-stacks collected from non-permeabilized HLMVECs are separated into apical and basal stacks and projected, the basal sum projections display signal only near the cell borders. No measurable basal GCX thickness is observed, as GCX thickness is quantified only above and below each nuclear body. Specifically, no signal is detected below the HLMVEC nuclei following WGA immunostaining without permeabilization (Supplementary Fig. 1). This suggests that without permeabilization WGA antibodies are not being adequately delivered to the basal HLMVEC GCX.

Most reports of the endothelial GCX have not included permeabilization during immunofluorescent staining, based on the long-standing assumption that the GCX exists solely on the luminal surface^11^. As shown in prior studies, staining with WGA in the absence of permeabilization labels only the apical GCX of confluent endothelial monolayers^12-14^. In contrast, when EC are tested with sucrose/saponin, the basal GCX can be successfully visualized^15^. 2% Sucrose and 0.1% saponin were used for membrane stabilization and permeabilization, respectively, over Triton-X100, a common detergent, as initial dose-dependent studies for basal GCX visualization displayed membrane discontinuities post permeabilization. The brightfield images revealed disruptions in the cell membrane, which were further confirmed by GCX staining in the orthogonal views (Supplementary Fig 2). These observations validate the membrane disruptions that occurred following permeabilization using Triton-X100. When comparing apical GCX signal intensity, quantified as the GCX signal above the z-stack plane corresponding to the centroid of the nuclei, between non-permeabilized HLMVECs and those permeabilized with 0.1% saponin, no significant differences were observed among HLMVECs exposed to FSS for the same duration (Fig. 1G–I). These results indicate that sucrose/saponin treatment preserves GCX integrity without significantly altering the apical signal (Fig 1). Subsequent data are grouped into apical and basal GCX signals, obtained from HLMVECs that were permeabilized after flow exposure and fixation. The collected z-stacks were separated using the described method to independently quantify apical and basal GCX metrics (Fig 6).

**Fig 1. Comparison of the apical glycocalyx (GCX) signal of permeabilized and non-permeabilized human lung microvascular endothelial cells (HLMVECs) exposed to 10 dynes/cm² of fluid shear stress revealed no statistically significant differences between HLMVECs of the same FSS exposure time.**

Sum projections (enface view of A-F) and accompanying orthogonal views (directly below enface view, A-F) of the apical GCX (green) and nuclei (blue) in non-permeabilized (A-C) and permeabilized (D-F) HLMVECs after static culture (A,D), 30 minutes (B,E), and 12 hours (C,F) of exposure to 10 dynes/cm^2^ fluid shear stress. No significant differences were found between GCX thickness (µm) (G), coverage (%) (H), and integrated intensity (fold-change (F.C.)) (I) of non-permeabilized and permeabilized HLMVECs.**”** – page 4-5 lines 95-144

**5.** Discussion Gaps:
**5.1**. There is a statement made that glycocalyx returns to baseline after 12 hr. The data shows 12 hr fluid shear stress leads to glycocalyx levels matching or falling below static conditions, depending on specific shear stress magnitude. This contradicts the expectation that fluid shear stress promotes glycocalyx growth and should be addressed.

**Response to Reviewer**

The authors appreciate this thoughtful observation and agree that the duration of FSS exposure could be extended to potentially observe further enrichment of the GCX, surpassing GCX enrichment observed after 3-4 days of static culture. We have added statements to the discussion highlighting this limitation. We have included suggestions for model development including longer FSS exposure durations, especially when further probing functional relevance, to improve physiological relevance of the data collected. However, here we sought to explore the bulk modulation of the basal GCX in response to FSS exposure, physiologically relevant FSS rates, and draw some conclusions about spatial temporal relationship of the GCX signal. As we observed disruption of the apical and basal GCX signal between static, 0.5 H, and 12 H, following reports of others reporting on one GCX element, a significant topological gradient in GCX signal, and a significant deviation in apical and basal metrics between the HLMVECS exposed different FSS rates by 12 hours of FSS exposure we concluded that 12 hours of FSS exposure was just for the comparisons to be drawn from the experiment.

Our current study was aimed at examining the cumulative modulation of the apical and basal GCX after exposure to physiologically relevant FSS rates to exposure the spatially dependent response of the GCX to FSS investigation. Previous studies have shown that GCX adaptation to fluid shear stress occurs over multiple timescales. We observed significant differences in apical and basal GCX signals between static, 0.5-hour, and 12-hour conditions, with clear shear-dependent modulation after 12 hours of exposure mirroring previous reports by Y. Zeng et al in 2014. We concluded that a 12-hour exposure period was sufficient for drawing initial comparisons while recognizing that extended FSS durations will be critical for future studies exploring GCX barrier functions. We have expanded the discussion to address these limitations and propose long term studies.

**Changes made to manuscript:**

“Future studies should extend the duration of FSS exposure to enable investigation of steady-state expression levels of GAGs) and proteoglycans, providing additional evidence for their physiological relevance in vivo.” - Page 14 lines 422-424

**5.2.** The authors should elaborate on the implications of the finding that very low fluid shear stress drives a decrease in glycocalyx coverage and intensity over time, as this has important disease relevance.

**Response to Reviewer**

We thank the reviewer for this valuable comment. We have elaborated on the physiological and pathological implications of the observed decrease in GCX coverage and intensity under very low fluid shear stress. Sustained low FSS has been shown to impair GCX integrity, increase endothelial permeability, and promote a pro-inflammatory, atheroprone phenotype. These findings align with our observations, suggesting that GCX degradation under low FSS contributes to endothelial dysfunction associated with vascular diseases such as atherosclerosis.

**Changes made to manuscript:**

“Pathologically low FSS rates, such as the 0.5 dynes/cm^2^ tested here, replicate shear stress rates found at vessel bifurcations and surrounding atherosclerotic plaques or vessel thrombosis^22-24^. Microvascular ECs exposed to FSS rates of 0.5 dynes/cm^2^ have been shown to produce elevated levels of inflammatory markers, indicating a pathological state or response to low FSS^25-29^. Literature reports indicate that when the GCX is exposed to pathophysiologically low FSS, its expression decreases compared to physiologically normal FSS^7,30,31^. This reduction is demonstrated through immunofluorescence staining, as well as mRNA and protein expression analyses of GCX core proteins and GAGs^7,30,31^ Our apical GCX results reflect these findings with apical GCX coverage, integrated intensity, and thickness trending up after FSS onset (Fig 3, Fig 5). The trend of decreasing expression is replicated in the basal GCX however, only after prolonged exposure. The combined findings of Thi et al. and Weinbaum et al., as previously discussed, suggest the existence of a torque threshold. If this threshold is not met due to low fluid shear stress (FSS) acting on the apical GCX, it does not lead to DPAB formation, stress fiber fragmentation, or basal focal adhesion remodeling^8,32^. We believe that these low resultant forces do not exceed the force threshold necessary to promote basal GCX shedding with apical FSS initiation rather, the delay observed in the basal GCX degradation could be due to excited inflammatory pathways resultant of the low FSS rather than the reaction force generated in the basal^27,29,30,33^. Apical GCX disruption due to low shear stress has been shown to increase EC permeability^30^. These low shear environments have also promote EC binding interactions with a variety of molecules and cells moving in blood flow such as cancer cells, immune cells, LDLs, and coagulation factors due to the reduction in EC barrier functions shear stress at the wall^2,4,5,12,22^. A more limited set of evidence has explored how these species are trafficked through the vessel once bound to the EC surface. The importance of junctional strength has been strongly emphasized along with the basal EC environment for trans endothelial migration^34-36^. Coupled with primary evidence previously discussed regarding how heparan sulfate gradients are generated across the EC in inflamed environments resulting in entrapment of chemokines in the basal ECs. The decrease in the bulk presence of both apical and basal GCX and the maintenance of the apical, basal GCX signal gradient observed here after prolonged exposure to physiologically low FSS may shed light on how EC permeability is dependent upon both apical and basal GCX presence and affected by pathological flow promoting migratory processes and the deposition of other small molecules (Fig 3, Fig 5) ^37-40^. Suggesting the role of the basal GCX, and its dynamic elements, in modulating EC permeability should be further explored as endothelium hyperpermeability is relevant in the development of many pathologies^30,41-44^.” - page 10-11 lines 307-337

**5.3.** The conclusion that basal glycocalyx is sensitive to apical fluid shear stress is intriguing but underexplored. The authors should expand on possible mechanisms and significance.

**Response to Reviewer**

We thank the reviewer for this thoughtful comment. We agree that the mechanisms underlying the sensitivity of the basal GCX to apical FSS were underexplored in the original manuscript. To address this, we have expanded the discussion to integrate proposed mechanisms and relevant literature describing how apical shear cues may be transduced to the basal GCX through cytoskeletal and membrane-coupled signaling pathways.

Specifically, studies have suggested that HS proteoglycans such as Syndecan-1 and Syndecan-4 serve as key mechanosensors and transmitters of shear-induced signals across the endothelial cell (Thi et al., Am J Physiol Heart Circ Physiol, 2004; Zeng & Tarbell, Front Cell Dev Biol, 2022). In the “bumper-car” model proposed by Tarbell and colleagues, HS chains deform under FSS, triggering intracellular signaling cascades through their cytoplasmic domains and associated adaptor proteins. These signals can propagate through the actin cytoskeleton to influence basal surface organization, focal adhesions, and extracellular matrix interactions, thereby potentially modulating the basal GCX.

The revised section now reads:

**Changes made to manuscript:**

“The in vitro flow conditions used in this study were designed to mimic the flow profiles typical of the vasculature. By examining EC and GCX responses under these shear environments, our findings provide insights into how local hemodynamic forces drive endothelial GCX response and gradients across the endothelium. Overall, when comparing apical and basal GCX metrics between groups of different FSS exposure we observed that apical and basal GCX metrics are significantly decreased in HLMVECs exposed to modulated FSS rates (0.5 and 30 dynes/cm^2^) compared to HLMVECs exposed to 10 dynes/cm^2^ (Fig 5). Relating to observations of increased endothelium permeability previously demonstrated in regions of modulated wall shear stress^2,4,5^. As GCX presence or abundance is correlated to barrier functions, our data suggest that the role of the basal GCX should be considered when investigating GCX and FSS dependent modulation of endothelium permeability functions^21,42,45,46^. Emerging evidence suggests that apical FSS can influence the basal GCX through cytoskeleton-mediated mechanotransduction. In the bumper-car model proposed by Thi et al. (2004), deformation of HS proteoglycans (Syndecan-1 and Syndecan-4) under shear stress initiates intracellular signaling that extends across the endothelial cell to the basal surface. Such coupling may explain how changes in apical shear can regulate basal GCX remodeling and basement membrane interactions^8^. We hypothesize that changes in signaling domain tension due to the differences in FSS experienced by our experimental groups, previously shown to regulate GAG transcription and apical GCX presence, may also regulate basal GCX presence and the difference observed when comparing groups across FSS magnitude.” – page 13 lines 377-395

**5.4.** The observation that differences between fluid shear stress magnitudes only emerge at 12 hours (Figure 5) suggests short-term studies may be inadequate for HLMVECs. This point deserves more emphasis in the discussion.

**Response to Reviewer**

The authors agree that the duration of FSS exposure could be extended to potentially observe further enrichment of the GCX, surpassing GCX enrichment observed after 3-4 days of static culture. We have added statements to the discussion highlighting this limitation. We have included suggestions for model development including longer FSS exposure durations, especially when further probing functional relevance, to improve physiological relevance of the data collected. However, here we sought to explore the bulk modulation of the basal GCX in response to FSS exposure, physiologically relevant FSS rates, and draw some conclusions about spatial temporal relationship of the GCX signal. As we observed disruption of the apical and basal GCX signal between static, 0.5 H, and 12 H, following reports of others reporting on one GCX element, a significant topological gradient in GCX signal, and a significant deviation in apical and basal metrics between the HLMVECS exposed different FSS rates by 12 hours of FSS exposure we concluded that 12 hours of FSS exposure was just for the comparisons to be drawn from the experiment.

We thank the reviewer for this valuable comment. We agree that extending the duration of FSS exposure could further elucidate GCX remodeling dynamics, potentially capturing later stages of GCX enrichment that may surpass those observed after 3–4 days of static culture. In response, we have expanded the Discussion to highlight this limitation and included suggestions for future model refinements, particularly the incorporation of longer FSS exposure periods to improve the physiological relevance and to assess the functional implications of GCX remodeling over time.

Our current study was designed to examine the bulk modulation of the basal GCX under physiologically relevant shear rates and to define the spatiotemporal relationship between apical and basal GCX responses. Previous studies have shown that GCX adaptation to shear stress occurs over multiple timescales—from early structural reorganization within hours to more stable proteoglycan enrichment after 24–48 hours (Y. Zeng et al 2014, J. X. Liu et al 2016). Consistent with these reports, we observed significant differences in apical and basal GCX signals between static, 0.5-hour, and 12-hour conditions, with clear shear-dependent modulation after 12 hours of exposure. Thus, we concluded that a 12-hour exposure period was sufficient for drawing initial comparisons while recognizing that extended FSS durations will be critical for future studies exploring long-term GCX adaptation and functionality.

**Changes made to manuscript:**

“Our recent review paper details research objectives necessary for addressing major gaps in our understanding of basal GCX function, specifically in the context of the ECs response to FSS^47^. These include probing specific GCX elements such as heparan sulfate, hyaluronic acid, and chondroitin sulfate to understand their presence in the basal GCX. The exact structural composition of basal proteoglycans and molecular weights of basal GCX GAGs should be investigated and compared to apical counterparts. Not only should the presence of individual GAGs and their core protein response to FSS be observed but the dependence of the basal GCX elements modulation in response to FSS on the presence of the apical GCX should be probed. For example, if the response of basal CD44 and hyaluronic acid to FSS were being examined, it would be important to understand if the effect of FSS exposure could be removed by enzymatic removal of the apical hyaluronic acid content or inhibition of actin polymerization. Experiments such as these would add to data previously presented models of endothelial FSS mechanotransduction and add to our understanding of endothelium permeability regulation through GCX. Future work should also address limitations of the data presented here. Extended the FSS exposure time would allow for investigation of steady state GAG and proteoglycan expression, providing further evidence of their presence in vivo. Functional studies should also characterize how the presence and modulation of basal GCX elements relates to the trans endothelial migration of immune and cancer cells as well as the trafficking of and macromolecules such as LDLs and cytokines key to disease progression. Similar to studies on apical GCX function, research on basal GCX function should also characterize basal GCX modulation and function in the EC response to other physiologically relevant forces in the basement membrane such as interstitial or transmural flow and substrate stiffness and composition. Ultimately studies should probe the basal GCX in vivo to examine basal GCX presence in both large and microvascular networks utilizing healthy and diseased models to shed more light on the basal GCX in relation to endothelium function.” – pages 13-14 lines 396-432

**6.** Goal vs. Interpretation Misalignment:
**6.1.** The results are heavily focused on permeabilization status, which does not clearly align with the stated goal of examining glycocalyx expression. The authors should clarify why this distinction is central to their analysis.

**Response to Reviewer**

We thank the reviewer for this important comment. We agree that clarification was needed regarding the relevance of permeabilization to our study’s aim of characterizing GCX expression. Traditionally, GCX investigations have been limited to the apical (luminal) surface of EC, with minimal consideration of potential basal GCX structures (I. Harding et al 2018, Y. Zeng et al 2014). However, because the antibodies used in this study needed to access both the apical and basal surfaces to visualize the full three-dimensional GCX network, permeabilization became a necessary methodological variable.

Our analysis of permeabilization status therefore serves not as the main research focus, but as an essential control to ensure accurate differentiation between apical and basal GCX localization and signal quantification. This distinction is central to validating our hypothesis that FSS modulates GCX composition both apically and basally through coordinated mechanotransduction. We have clarified this rationale in the revised Methods and Results sections.

**Changes made to manuscript:**

“HLMVECs were confirmed to produce a robust apical GCX in response to physiologically normal FSS by probing the GCX following methods previously used to visualize and quantify the luminal GCX (Supplementary Fig 1). After exposure to 10 dynes/cm² of FSS for 12 hours, HLMVECs were fixed as described and their GCX was tagged without permeabilization to tag only GCX components naturally exposed to antibodies during the immunostaining process. Full thickness z-stacks were not separated using the apical / basal image separate process described, instead entire z-stack collected was sum projected and quantified. Metrics of coverage and integrated intensity were found to significantly increase compared to static conditions (p = 0.0014, p = 0.0135 respectively), and the levels observed after 30 minutes of exposure (p = 0.0002, p = 0.0113 respectively) (Supplementary Fig 1). This indicates a sustained and overall enhancement in GCX expression in response to prolonged shear stress. Measurements of GCX thickness (µm) correspond with the literature ranges reported (Supplementary Fig 1)^12,16,17^. These results align with what has been reported for prominent GCX components in other endothelial cell types, such as HUVECs when using the typically presented method of GCX staining, visualization, and quantification^14,18-21^. It is also important to note that when the z-stacks collected of non-permeabilized HLMVECs are separated into apical and basal z-stacks and projection, basal sum projections only display signal near cell boarders and no basal GCX thickness is measurable as GCX thickness is only quantified above and below each nuclear body as no signal is observed below the HLMVEC nuclei after WGA immunostaining without permeabilization (Supplementary Fig. 1). This suggests without permeabilization WGA antibodies are not being adequately delivered to the basal HLMVEC GCX.

Most reports of the endothelial GCX have not included permeabilization during immunofluorescent staining, based on the long-standing assumption that the GCX exists solely on the luminal surface^11^. As shown in prior studies, staining with WGA in the absence of permeabilization labels only the apical GCX of confluent endothelial monolayers^12-14^. In contrast, when EC are tested with sucrose/saponin, the basal GCX can be successfully visualized^15^. 2% Sucrose and 0.1% saponin were used for membrane stabilization and permeabilization, respectively, over Triton-X100, a common detergent, as initial dose-dependent studies for basal GCX visualization displayed membrane discontinuities post permeabilization. The brightfield images revealed disruptions in the cell membrane, which were further confirmed by GCX staining in the orthogonal views (Supplementary Fig 2). These observations validate the membrane disruptions that occurred following permeabilization using Triton-X100. When comparing metrics quantifying apical GCX signal or the GCX signal above the z-stack plane that corresponds to the centroid of the nuclei in the field of view between non-permeabilized HLMVECs and those permeabilized with 0.1% saponin, no significant differences were observed between HLMVECs exposed to FSS for the same duration (Fig 1G-I). These results indicate that sucrose/saponin treatment preserves GCX integrity without significantly altering the apical signal (Fig 1). Further data presented is grouped by apical and basal GCX signal derived from permeabilizing HLMVECs post flow exposure and fixation and separating the z-stack collected using the separation method described to quantify apical and basal metrics separately (Figure 6).

**Fig 1. Comparison of the apical glycocalyx (GCX) signal of permeabilized and non-permeabilized human lung microvascular endothelial cells (HLMVECs) exposed to 10 dynes/cm² of fluid shear stress revealed no statistically significant differences between HLMVECs of the same FSS exposure time.**

Sum projections (enface view of A-F) and accompanying orthogonal views (directly below enface view, A-F) of the apical GCX (green) and nuclei (blue) in non-permeabilized (A-C) and permeabilized (D-F) HLMVECs after static culture (A,D), 30 minutes (B,E), and 12 hours (C,F) of exposure to 10 dynes/cm^2^ fluid shear stress. No significant differences were found between GCX thickness (µm) (G), coverage (%) (H), and integrated intensity (fold-change (F.C.)) (I) of non-permeabilized and permeabilized HLMVECs.

After HLMVECs were exposed to 10 dynes/cm^2^ for 0.5 or 12 hours the apical and basal GCX was tagged, imaged, separated into apical and basal signal, and the apical and basal GCX signal was quantified in terms of integrated intensity, coverage (&), and thickness (µm). When comparing GCX thickness, basal GCX thickness (µm) decreased significantly after 30 minutes and 12 hours of exposure (p = 0.0138, p < 0.0001 respectively) (Fig 2G). Apical GCX thickness (µm) increased slightly with FSS exposure, however, insignificantly. Changes in GCX thickness (µm) can be seen in orthogonal views accompanying sum projections in Fig 2A-F. Apical GCX (Fig 2A-C) coverage decreased significantly after 30 minutes of exposure to 10 dynes/cm^2^ (p = 0.0205), while both apical GCX coverage and integrated intensity (Fig 2H and Fig 2I, respectively) significantly increased between 30 minutes and 12 hours of exposure (p < 0.0001 and p = 0.0038 respectively). Apical GCX expression metrics show an initial GCX disturbance with FSS onset and recovery with extended exposure, as seen in the decrease in coverage and integrated intensity after 30 minutes, followed by a recovery to near static values after 12 hours of exposure to FSS (Fig 2H-I: green). The basal GCX also displayed this disturbance. Basal GCX (Fig 2D-F) integrated intensity (Fig 2I: purple) was found to significantly decrease after 30 minutes of exposure to 10 dynes/cm^2^ of FSS, recovering to approximately static levels after 12 hours of exposure (p < 0.0001). Basal GCX coverage (Fig 2H: purple) did not vary significantly. Basal GCX coverage and integrated intensity remained significantly higher than apical GCX measures, while inversely, basal GCX thickness remained significantly lower than apical GCX thickness (Fig 2H-I). These relationships were present in all FSS rates tested, aligning with previous observations of a heparan sulfate gradient across the endothelium (Fig 3 and Fig 4)^15^.” –pages 4-7 lines 95-165

**6.2.** Several findings appear to reconfirm prior published work. The authors should clarify what is novel in their observations and how their data extend or challenge existing literature.

**Response to Reviewer**

We thank the reviewer for this thoughtful comment. While our findings align with previous reports describing the sensitivity of the apical GCX to FSS (Y. Zeng et al 2014, Thi et al. 2004), our study provides novel insights into the basal GCX, a region that has been largely uncharacterized in literature. Specifically, we demonstrate for the first time that apical FSS cues elicit distinct and coordinated remodeling responses in both apical and basal GCX domains.

We hope that statements added throughout the manuscript have now clarified this, specifically we added this statement to the discussion to conclude findings:

**Changes made to manuscript:**

“In summary, using optimized fixation and permeabilization methods, we were able to visualize and quantify the basal GCX signal under physiologically relevant shear conditions, revealing time- and shear-dependent modulation not previously reported. These results suggest that the basal GCX is sensitive to apical FSS, exhibiting distinct expression patterns compared to the apical GCX and showing higher overall expression. Our results extend existing models of GCX mechanotransduction by suggesting that apical shear forces may be transduced through cytoskeletal coupling mediated signaling pathways to regulate basal GCX organization and presence^8^. This dual-surface analysis advances current understanding of endothelial mechanobiology by linking apical flow sensing to basal matrix interactions, thereby providing a more complete picture of GCX-mediated endothelial function. This may suggest that for disease with primary development in the vascular bed or relation to vascular permeability, the basal GCX and its effect on EC barrier function and mechanotransduction should be considered along with apical function^45,46^.” – page 13 lines 396-408

**Reviewer #2:** 
**1.** The manuscript lacks sufficient details in the methodology, for example:
Line 348: Need source for 3-4 days being sufficient for full maturation of GCX on HLMVECs
Line 364 Please provide source which shows that WGA binds to ‘all lectins of the GCX’

**Response to Reviewer**

We thank the reviewer for this helpful comment. We limited static culture of HLMVECs to three to four days, as this period consistently produces a continuous, confluent endothelial monolayer with a robust GCX signal, while avoiding the tradeoffs associated with prolonged static maturation. Extended culture durations often necessitate lower seeding densities to prevent overconfluence and subsequent HLMVEC detachment or shedding upon initiation of flow, which could introduce heterogeneity in cell age and GCX development.

This 3–4-day static window aligns with prior reports demonstrating that 3–5 days of culture are sufficient for endothelial confluence and GCX establishment before flow exposure (Giantsos-Adams KM et al. 2013, Zeng Y. et al 2013). Moreover, our data show that this culture period yields a well-developed GCX, as evidenced by a clear apical GCX disruption at 0.5 hours of flow initiation and recovery to static levels after 12 hours of physiological FSS (10 dynes/cm²), a pattern consistent with GCX adaptation kinetics reported in the literature (Y. Zeng, 2014).

Regarding the reviewer’s second point, we have clarified that wheat germ agglutinin (WGA) is a lectin that binds to sialic acid and N-acetylglucosamine (GlcNAc) residues—key glycan components distributed across multiple GCX proteoglycans and glycoproteins (Reitsma et al, 2007, Harding IC et al, 2018).

**Changes made to manuscript:**

“WGA binds broadly to sialic acid- and GlcNAc-containing moieties within the GCX.” – line 455-456

**2.** Line 346 Can the authors elaborate on why media that is optimized for breast tissue is being used for lung endothelial cells?

**Response to Reviewer**

We thank the reviewer for this helpful comment. We acknowledge the confusion caused by the description of the growth medium. Although “MEGM” is sometimes associated with mammary endothelial cell media, the formulation provided by Cell Applications Inc. is specifically optimized for human microvascular endothelial cells (HLMVECs) and is recommended by the supplier for use with this cell type. We have clarified this in the revised Methods section to avoid ambiguity and confirm that the medium used is appropriate and validated for HLMVEC culture.

**Changes made to manuscript:**

“HLMVECs (Cell Applications Inc.) were cultured and maintained in microvascular endothelial cell growth medium (MEGM; Cell Applications Inc.).” – page 14 lines 435-436

**3.** Line 365 the use of streptavidin is confusing, as it is unclear where biotin is used in the methods

**Response to Reviewer**

We thank the reviewer for this helpful comment and apologize for the confusion. The WGA used in this study is biotinylated, which enables its subsequent detection with streptavidin-conjugated fluorophores. This has been clarified in the Methods section.

**Changes made to manuscript:**

“Samples were allowed to incubate in primary solution containing biotinylated wheat germ agglutinin (WGA) (1:500, Vector) in 1% BSA in PBS overnight. WGA was utilized as it binds to all lectins of the GCX^11,13,15^. Negative controls were generated by omitting WGA in the primary solution. The secondary detection solution contained Streptavidin Alexa Fluor 488 (1:1000, Invitrogen) in 1% BSA in PBS. Samples were mounted with Fluoromount-G with DAPI mounting media for immunofluorescent nuclei tagging (Invitrogen).” – page 15 lines 453-459

**4.** Line 389 The authors state that: “Background intensity was determined to be the intensity that corresponds to 6% of the normalized distribution of pixel intensities in the apical GCX and 8% in the basal GCX for each experimental group” Can the authors elaborate on how these 6 and 8 % values were determined.

**Response to Reviewer**

We thank the reviewer for this insightful question and apologize for any lack of clarity. The 6% (apical) and 8% (basal) background intensity thresholds were derived from negative control samples, which represent the average non-specific fluorescence signal in each imaging plane. Specifically, we measured the average field-of-view intensity from apical and basal sum projections of negative controls and compared these values to the maximum possible pixel intensity within each image stack. This analysis revealed that background fluorescence corresponded to approximately 6% and 8% of the maximum (fully saturated) intensity for the apical and basal GCX images, respectively.

**Changes made to manuscript:**

“Background intensity was determined to be 6% and 8% of the normalized distribution of pixel intensities in the apical and basal GCX, respectively, corresponding to the average integrated intensity measured from apical and basal negative control samples.” – page 16-17 lines 506- 509

**5.** The manuscript lacks depth as the same assessments were done across different conditions and turned into separate figures. The different FSS rates could be combined into a single figure. This would leave room for figures that focus on specific glycan changes under FSS conditions.

**Response to Reviewer**

We thank the reviewer for this helpful comment. We agree that the presentation of multiple figures could initially appear repetitive; however, the data were intentionally organized this way to enable distinct statistical comparisons and to highlight different biological interpretations.

In Figures 2–4, data are grouped by apical vs. basal regions and exposure time for each FSS rate. This structure allows for two-way ANOVA comparisons both between apical and basal groups and across exposure times for each GCX metric (coverage, integrated intensity, and thickness).

In contrast, Figure 5 groups data by FSS rate and exposure time, enabling a second level of analysis comparing GCX metrics across FSS rates at each time point and across time within a single FSS rate. Together, these complementary analyses capture both within-rate (temporal and spatial) and between-rate (magnitude-dependent) GCX responses.

We have clarified this distinction in the Statistical Comparisons section and emphasized in the text how Figures 2–4 and Figure 5 collectively provide a comprehensive view of GCX modulation under varying FSS conditions.

**Changes made to manuscript:**

“Each experimental sample is represented as one set of apical and basal metrics (integrated intensity, coverage, and thickness) such that the metric can be compared between apical and basal, across exposure times (apical 0.5-hour metrics vs. apical 12-hour metrics), and with other apical and basal metrics after exposure to a different FSS rate (basal 12-hour metrics: 10 dynes/cm^2^ vs 0.5 dynes/cm^2^). All qualitative data is represented by the mean of the group with variance presented as the standard error of the mean generated by at least five independent experiments (numerical replicates subsequently listed for each experimental group in Results). Multi-comparison one-way ANOVA tests with Brown-Forsythe and Welch parameters were performed to determine statistical significance differences between apical and basal means at each timepoint, and across apical and basal means independently when comparing GCX expression metrics over FSS exposure time within one shear rate. A two-way grouped ANOVA test was run to determine the effects of FSS rate and time on each GCX metric. P-values were considered significant if less than 0.05. All resultant P-values can be found in Supplementary Table 1. All statics and data plots were generated using GraphPad Prism 10 (version 6.04 for Windows, GraphPad Software, [www.graphpad.com)](http://www.graphpad.com)).” – page 17 lines 524 - 537

**6.** The manuscript could benefit from the addition of a lectin with specificity to a particular glycan component of the GCX (e.g., hyaluronan, or heparan sulfates). The use of a Pan-GCX marker does imply the change, but the mechanism of those changes could be parsed out with more specific markers for GCX components. This was mentioned on line 334, but at least some data should be presented showing specific changes in a particular glycan after FSS.

We thank the reviewer for this insightful comment and agree that incorporating lectins or antibodies with specificity for individual GCX components, such as hyaluronan or heparan sulfate, would provide valuable mechanistic detail. However, this level of molecular specificity is beyond the scope of the current study, which focuses on establishing and validating a methodology to distinguish apical and basal GCX responses under defined FSS conditions using a pan-GCX marker (WGA).

That said, our ongoing work and preliminary data indicate differential expression of specific glycosaminoglycans (including hyaluronan) under varying FSS magnitudes, supporting the feasibility and importance of this next step. We have added a statement in the discussion acknowledging this limitation and outlining future directions that will use glycan-specific markers to further elucidate the molecular mechanisms of GCX remodeling.

**Changes made to manuscript:**

“Our recent review paper details research objectives necessary for addressing major gaps in our understanding of basal GCX function, specifically in the context of the ECs response to FSS^47^. These include probing specific GCX elements such as heparan sulfate, hyaluronic acid, and chondroitin sulfate to understand their presence in the basal GCX. The exact structural composition of basal proteoglycans and molecular weights of basal GCX GAGs should be investigated and compared to apical counterparts. Not only should the presence of individual GAGs and their core protein response to FSS be observed but the dependence of the basal GCX elements modulation in response to FSS on the presence of the apical GCX should be probed. For example, if the response of basal CD44 and hyaluronic acid to FSS were being examined, it would be important to understand if the effect of FSS exposure could be removed by enzymatic removal of the apical hyaluronic acid content or inhibition of actin polymerization. Experiments such as these would add to data previously presented models of endothelial FSS mechanotransduction and add to our understanding of endothelium permeability regulation through the GCX. Future work should also address limitations of the data presented here. Extended the FSS exposure time would allow for investigation of steady state GAG and proteoglycan expression, providing further evidence of their presence in vivo. Functional studies should also characterize how the presence and modulation of basal GCX elements relates to the trans endothelial migration of immune and cancer cells as well as the trafficking of and macromolecules such as LDLs and cytokines key to disease progression. Similar to studies on apical GCX function, research on basal GCX function should also characterize basal GCX modulation and function in the EC response to other physiologically relevant forces in the basement membrane such as interstitial or transmural flow and substrate stiffness and composition. Ultimately studies should probe the basal GCX in vivo to examine basal GCX presence in both large and microvascular networks utilizing healthy and diseased models to shed more light on the basal GCX in relation to endothelium function.” –– pages 13-14 lines 396-432

**7.** In the Introduction (line 43) and in the discussion, there are mentions of heparan sulfates and syndecan-1. These markers could have been assessed in this model system using validated probes for their expression.
**Response to Reviewer**

We thank the reviewer for highlighting this potential. The authors agree that additional lectin staining, isolating individual GAG components and their proteoglycans response to FSS is a necessary follow up to the work presented here demonstrating the bulk apical and basal GCX response to FSS exposure. We agree that these experiments should also probe further timepoints and delve into the mechanistic regulation of the basal GCX as also discussed during the review. We have expanded upon the hypothesis and future work required while specifying the knowledge gained to better state the novel results we have demonstrated while emphasizing what is left to be done. However, we do not believe that level of GAG specificity fits with the outlined work here. A statement has been added to the discussion outlining study limitations and future work.

**Changes made to manuscript:**

“Our recent review paper details research objectives necessary for addressing major gaps in our understanding of basal GCX function, specifically in the context of the ECs response to FSS^47^. These include probing specific GCX elements such as heparan sulfate, hyaluronic acid, and chondroitin sulfate to understand their presence in the basal GCX. The exact structural composition of basal proteoglycans and molecular weights of basal GCX GAGs should be investigated and compared to apical counterparts. Not only should the presence of individual GAGs and their core protein response to FSS be observed but the dependence of the basal GCX elements modulation in response to FSS on the presence of the apical GCX should be probed. For example, if the response of basal CD44 and hyaluronic acid to FSS were being examined, it would be important to understand if the effect of FSS exposure could be removed by enzymatic removal of the apical hyaluronic acid content or inhibition of actin polymerization. Experiments such as these would add to data previously presented models of endothelial FSS mechanotransduction and add to our understanding of endothelium permeability regulation through the GCX. Future work should also address limitations of the data presented here. Extended the FSS exposure time would allow for investigation of steady state GAG and proteoglycan expression, providing further evidence of their presence in vivo. Functional studies should also characterize how the presence and modulation of basal GCX elements relates to the trans endothelial migration of immune and cancer cells as well as the trafficking of and macromolecules such as LDLs and cytokines key to disease progression. Similar to studies on apical GCX function, research on basal GCX function should also characterize basal GCX modulation and function in the EC response to other physiologically relevant forces in the basement membrane such as interstitial or transmural flow and substrate stiffness and composition. Ultimately studies should probe the basal GCX in vivo to examine basal GCX presence in both large and microvascular networks utilizing healthy and diseased models to shed more light on the basal GCX in relation to endothelium function.” – pages 13-14 lines 396-432

**8.** Can the authors elaborate on how changes in cell polarity (i.e., movement of the nucleus, as demarcated by DAPI) could influence their interpretation of Apical vs. Basal surfaces under different flow conditions?
**Response to Reviewer**

We thank the reviewer for this thoughtful comment. We agree that cell polarity, particularly the reorientation of the nucleus under flow, could potentially influence the interpretation of apical versus basal image separation. To address this, during development of our apical/basal separation pipeline, we analyzed both XZ and YZ orthogonal views to measure the Z-centroid of each nucleus across conditions. We typically captured ~20 nuclei per field of view and found no significant difference in Z-centroid measurements between XZ and YZ views for cells exposed to 10 dynes/cm² FSS. This confirms that the nuclear centroid measurement, and therefore our z-slice segmentation was not affected by cell elongation or polarity changes following flow exposure.

To improve efficiency without compromising accuracy, subsequent analyses used only the XZ orthogonal view, which aligns with the flow direction and significantly reduced processing time. We have clarified this methodology in the text:

**Changes made to manuscript:**

“Initially both the XZ and YZ orthogonal view of each nucleus was analyzed to measure the Z-centroid of each nucleus across conditions. We typically captured ~20 nuclei per field of view and found no significant difference in Z-centroid measurements between XZ and YZ views for cells exposed to 10 dynes/cm² FSS. This confirms that the nuclear centroid measurement, and therefore our z-slice segmentation was not affected by cell elongation or polarity changes following flow exposure. To improve efficiency without compromising accuracy, subsequent analyses used only the XZ orthogonal view, which aligns with the flow direction and significantly reduced processing time. Therefore, the Z-stack slice that corresponds to the average height of all nuclei centroids, as measured in the XZ plane, was used to divide the original stack into apical and basal sub-stacks following methods previously presented for EC vertical spatial distribution analysis as seen and described in Figure 6.” – page 15-16 lines 470 - 483

**9.** Line 225: The use of the term peptides here seems vague. Please be more specific in the discussion.

**Response to Reviewer**

We thank the reviewer for this constructive comment. We agree that the original phrasing was vague, and we have revised the discussion to provide greater specificity. The text now reads:

**Changes made to manuscript:**

“Circulating peptides and enzymes such as atrial natriuretic peptide and heparanase are known to increase in circulation during ischemia and reperfusion injury. These molecules, often referred to as ‘shedases,’ mediate rapid GCX degradation by either promoting syndecan-1 ectodomain shedding or by enzymatically cleaving heparan sulfate chains from their core proteins (heparanase), leading to acute GCX loss and endothelial dysfunction.” – page 9 lines 267-272

**10.** Line 363 Should Wheat germ albumin be ‘agglutinin’?

**Response to Reviewer**

Yes, updated in text.

**11.** Line 360 Bovine serum albumin (BSA) needs to be defined earlier

**Response to Reviewer**

Updated in text.

**12.** Figure legends should be placed at the end of the manuscript on initial submission, rather than embedded in the results section.

**Response to Reviewer**

We thank the reviewer for this comment. The figure legends were not relocated, as the PLOS ONE manuscript guidelines specify that “each figure caption should be inserted immediately after the first paragraph in which it is cited in the article file.” Accordingly, we have retained the current placement of figure legends to ensure compliance with the journal’s formatting requirements.

1.	Dai G, Kaazempur-Mofrad MR, Natarajan S, Zhang Y, Vaughn S, Blackman BR, Kamm RD, García-Cardeñ G, Gimbrone MA. Distinct endothelial phenotypes evoked by arterial waveforms derived from atherosclerosis-susceptible and-resistant regions of human vasculature. In; 2004.

2.	Zhang C, Xie S, Li S, Pu F, Deng X, Fan Y, Li D. Flow patterns and wall shear stress distribution in human internal carotid arteries: the geometric effect on the risk for stenoses. *J Biomech*. 2012;45:83-89. doi: 10.1016/j.jbiomech.2011.10.001

3.	Younis HF, Kaazempur-Mofrad MR, Chan RC, Isasi AG, Hinton DP, Chau AH, Kim LA, Kamm RD. Hemodynamics and wall mechanics in human carotid bifurcation and its consequences for atherogenesis: investigation of inter-individual variation. *Biomech Model Mechanobiol*. 2004;3:17-32. doi: 10.1007/s10237-004-0046-7

4.	Chen L, Qu H, Liu B, Chen B-C, Yang Z, Shi D-Z, Zhang Y. Low or oscillatory shear stress and endothelial permeability in atherosclerosis. *Frontiers in Physiology*. 2024;15. doi: 10.3389/fphys.2024.1432719

5.	Lawrence MB, Kansas GS, Kunkel EJ, Ley K. Threshold levels of fluid shear promote leukocyte adhesion through selectins (CD62L,P,E). *J Cell Biol*. 1997;136:717-727. doi: 10.1083/jcb.136.3.717

6.	Zhang J, Kong X, Wang Z, Gao X, Ge Z, Gu Y, Ye P, Chao Y, Zhu L, Li X, et al. AMP-activated protein kinase regulates glycocalyx impairment and macrophage recruitment in response to low shear stress. *Faseb j*. 2019;33:7202-7212. doi: 10.1096/fj.201801869RRR

7.	Yang H, Zhu L, Chao Y, Gu Y, Kong X, Chen M, Ye P, Luo J, Chen S. Hyaluronidase2 (Hyal2) modulates low shear stress-induced glycocalyx impairment via the LKB1/AMPK/NADPH oxidase-dependent pathway. *J Cell Physiol*. 2018;233:9701-9715. doi: 10.1002/jcp.26944

8.	Thi MM, Tarbell JM, Weinbaum S, Spray DC. The role of the glycocalyx in reorganization of the actin cytoskeleton under fluid shear stress: A ''bumper-car'' model. *PNAS November*. 2004;23:16483-16488.

9.	Baeyens N, Mulligan-Kehoe MJ, Corti F, Simon DD, Ross TD, Rhodes JM, Wang TZ, Mejean CO, Simons M, Humphrey J, et al. Syndecan 4 is required for endothelial alignment in flow and atheroprotective signaling. *Proceedings of the National Academy of Sciences of the United States of America*. 2014;111:17308-17313. doi: 10.1073/pnas.1413725111

10.	Liu X, Fan Y, Deng X. Mechanotransduction of flow-induced shear stress by endothelial glycocalyx fibers is torque determined. *ASAIO Journal*. 2011;57:487-494. doi: 10.1097/MAT.0b013e318233b5ed

11.	Reitsma S, Slaaf DW, Vink H, Van Zandvoort MAMJ, Oude Egbrink MGA. The endothelial glycocalyx: Composition, functions, and visualization. In: *Pflugers Archiv European Journal of Physiology*. 2007:345-359.

12.	Mensah SA, Nersesyan AA, Harding IC, Lee CI, Tan X, Banerjee S, Niedre M, Torchilin VP, Ebong EE. Flow-regulated endothelial glycocalyx determines metastatic cancer cell activity. *FASEB Journal*. 2020;34:6166-6184. doi: 10.1096/fj.201901920R

13.	Harding IC, Mitra R, Mensah SA, Herman IM, Ebong EE. Pro-atherosclerotic disturbed flow disrupts caveolin-1 expression, localization, and function via glycocalyx degradation. *Journal of Translational Medicine*. 2018;16. doi: 10.1186/s12967-018-1721-2

14.	Zeng Y, Tarbell JM. The adaptive remodeling of endothelial glycocalyx in response to fluid shear stress. *PLoS ONE*. 2014;9. doi: 10.1371/journal.pone.0086249

15.	Stoler-Barak L, Moussion C, Shezen E, Hatzav M, Sixt M, Alon R. Blood vessels pattern heparan sulfate gradients between their apical and basolateral aspects. *PLoS ONE*. 2014;9. doi: 10.1371/journal.pone.0085699

16.	Gao L, Lipowsky HH. Composition of the endothelial glycocalyx and its relation to its thickness and diffusion of small solutes. *Microvascular Research*. 2010;80:394-401. doi: 10.1016/j.mvr.2010.06.005

17.	Bai K, Wang W. Shear stress-induced redistribution of the glycocalyx on endothelial cells in vitro. *Biomechanics and Modeling in Mechanobiology*. 2014;13:303-311. doi: 10.1007/s10237-013-0502-3

18.	Giantsos-Adams KM, Koo AJA, Song S, Sakai J, Sankaran J, Shin JH, Garcia-Cardena G, Dewey CF. Heparan sulfate regrowth profiles under laminar shear flow following enzymatic degradation. *Cellular and Molecular Bioengineering*. 2013;6:160-174. doi: 10.1007/s12195-013-0273-z

19.	Mensah SA, Cheng MJ, Homayoni H, Plouffe BD, Coury AJ, Ebong EE. Regeneration of glycocalyx by heparan sulfate and sphingosine 1-phosphate restores interendothelial communication. *PLoS ONE*. 2017;12. doi: 10.1371/journal.pone.0186116

20.	Psefteli PM, Kitscha P, Vizcay G, Fleck R, Chapple SJ, Mann GE, Fowler M, Siow RC. Glycocalyx sialic acids regulate Nrf2-mediated signaling by fluid shear stress in human endothelial cells. *Redox Biology*. 2021;38. doi: 10.1016/j.redox.2020.101816

21.	Gouverneur M, Spaan JAE, Pannekoek H, Fontijn RD, Vink H. Fluid shear stress stimulates incorporation of hyaluronan into endothelial cell glycocalyx. *Am J Physiol Heart Circ Physiol*. 2006;290:458-462. doi: 10.1152/ajpheart.00592.2005.-Vascular

22.	Moerman AM, Korteland S, Dilba K, van Gaalen K, Poot DHJ, van Der Lugt A, Verhagen HJM, Wentzel JJ, van Der Steen AFW, Gijsen FJH, et al. The Correlation Between Wall Shear Stress and Plaque Composition in Advanced Human Carotid Atherosclerosis. *Front Bioeng Biotechnol*. 2021;9:828577. doi: 10.3389/fbioe.2021.828577

23.	Urschel K, Tauchi M, Achenbach S, Dietel B. Investigation of wall shear stress in cardiovascular research and in clinical practice—from bench to bedside. In: *International Journal of Molecular Sciences*. 2021.

24.	Kwak BR, Bäck M, Bochaton-Piallat ML, Caligiuri G, Daemen MJ, Davies PF, Hoefer IE, Holvoet P, Jo H, Krams R, et al. Biomechanical factors in atherosclerosis: mechanisms and clinical implications. *Eur Heart J*. 2014;35:3013-3020, 3020a-3020d. doi: 10.1093/eurheartj/ehu353

25.	Dabagh M, Jalali P, Butler PJ, Randles A, Tarbell JM. Mechanotransmission in endothelial cells subjected to oscillatory and multi-directional shear flow. *Journal of the Royal Society Interface*. 2017;14. doi: 10.1098/rsif.2017.0185

26.	He G, Gao Y, Feng L, He G, Wu Q, Gao W, Lin L, Wang W. Correlation Between Wall Shear Stress and Acute Degradation of the Endothelial Glycocalyx During Cardiopulmonary Bypass. *Journal of Cardiovascular Translational Research*. 2020;13. doi: 10.1007/s12265-020-10027-2

27.	Huang H, Ren P, Zhao Y, Weng H, Jia C, Yu F, Nie Y. Low shear stress induces inflammatory response via CX3CR1/NF-kappaB signal pathway in human umbilical vein endothelial cells. *Tissue Cell*. 2023;82:102043. doi: 10.1016/j.tice.2023.102043

28.	Ishibazawa A, Nagaoka T, Takahashi T, Yamamoto K, Kamiya A, Ando J, Yoshida A. Effects of shear stress on the gene expressions of endothelial nitric oxide synthase, endothelin-1, and thrombomodulin in human retinal microvascular endothelial cells. *Invest Ophthalmol Vis Sci*. 2011;52:8496-8504. doi: 10.1167/iovs.11-7686

29.	Ishibazawa A, Nagaoka T, Yokota H, Ono S, Yoshida A. Low shear stress up-regulation of proinflammatory gene expression in human retinal microvascular endothelial cells. *Exp Eye Res*. 2013;116:308-311. doi: 10.1016/j.exer.2013.10.001

30.	Chen L, Qu H, Liu B, Chen BC, Yang Z, Shi DZ, Zhang Y. Low or oscillatory shear stress and endothelial permeability in atherosclerosis. *Front Physiol*. 2024;15:1432719. doi: 10.3389/fphys.2024.1432719

31.	Lin L, Gao W, Feng L, Wang C, Yang R, Wang W, Wu Q. Autophagy Induced by Low Shear Stress Leads to Endothelial Glycocalyx Disruption. *J Vasc Res*. 2024;61:77-88. doi: 10.1159/000537772

32.	Weinbaum S, Zhang X, Han Y, Vink H, Cowin SC. Mechanotransduction and flow across the endothelial glycocalyx. *Proc Natl Acad Sci U S A*. 2003;100:7988-7995. doi: 10.1073/pnas.1332808100

33.	Kong X, Chen L, Ye P, Wang Z, Zhang J, Ye F, Chen S. The role of HYAL2 in LSS-induced glycocalyx impairment and the PKA-mediated decrease in eNOS–Ser-633 phosphorylation and nitric oxide production. *Molecular Biology of the Cell*. 2016;27:3972-3979. doi: 10.1091/mbc.E16-04-0241

34.	Sekiguchi R, Yamada KM. Basement Membranes in Development and Disease. *Current Topics in Developmental Biology*. 2018;130:143-191. doi: 10.1016/bs.ctdb.2018.02.005

35.	Alon R, van Buul JD. Leukocyte Breaching of Endothelial Barriers: The Actin Link. *Trends in Immunology*. 2017;38:606-615. doi: <https://doi.org/10.1016/j.it.2017.05.002>

36.	Kelley LC, Lohmer LL, Hagedorn EJ, Sherwood DR. Traversing the basement membrane in vivo: a diversity of strategies. *J Cell Biol*. 2014;204:291-302. doi: 10.1083/jcb.201311112

37.	Armstrong SM, Sugiyama MG, Fung KYY, Gao Y, Wang C, Levy AS, Azizi P, Roufaiel M, Zhu SN, Neculai D, et al. A novel assay uncovers an unexpected role for SR-BI in LDL transcytosis. *Cardiovascular Research*. 2015;108:268-277. doi: 10.1093/cvr/cvv218

38.	Ghim M, Alpresa P, Yang S-W, Braakman ST, Gray SG, Sherwin SJ, Reeuwijk Mv, Weinberg PD. Visualization of three pathways for macromolecule transport across cultured endothelium and their modification by flow. *Am J Physiol Heart Circ Physiol*. 2017:959-973.

39.	Ghaffari S, Nabi FN, Sugiyama MG, Lee WL. Estrogen inhibits LDL (low-density lipoprotein) transcytosis by human coronary artery endothelial cells via GPER (G-protein-coupled estrogen receptor) and SR-BI (scavenger receptor class B type 1). *Arteriosclerosis, Thrombosis, and Vascular Biology*. 2018;38:2283-2294. doi: 10.1161/ATVBAHA.118.310792

40.	Huang L, Chambliss KL, Gao X, Yuhanna IS, Behling-Kelly E, Bergaya S, Ahmed M, Michaely P, Luby-Phelps K, Darehshouri A, et al. SR-B1 drives endothelial cell LDL transcytosis via DOCK4 to promote atherosclerosis. *Nature*. 2019;569:565-569. doi: 10.1038/s41586-019-1140-4

41.	Abassi Z, Armaly Z, Heyman SN. Glycocalyx Degradation in Ischemia-Reperfusion Injury. *Am J Pathol*. 2020;190:752-767. doi: 10.1016/j.ajpath.2019.08.019

42.	Becker BF, Jacob M, Leipert S, Salmon AHJ, Chappell D. Degradation of the endothelial glycocalyx in clinical settings: Searching for the sheddases. *British Journal of Clinical Pharmacology*. 2015;80. doi: 10.1111/bcp.12629

43.	Chelazzi C, Villa G, Mancinelli P, De Gaudio AR, Adembri C. Glycocalyx and sepsis-induced alterations in vascular permeability. In: *Critical Care*. BioMed Central Ltd.; 2015.

44.	Jin J, Fang F, Gao W, Chen H, Wen J, Wen X, Chen J. The Structure and Function of the Glycocalyx and Its Connection With Blood-Brain Barrier. In: *Frontiers in Cellular Neuroscience*. Frontiers Media S.A.; 2021.

45.	Curry FE, Adamson RH. Endothelial glycocalyx: Permeability barrier and mechanosensor. In: *Annals of Biomedical Engineering*. 2012:828-839.

46.	Gouverneur M, Van Den Berg B, Nieuwdorp M, Stroes E, Vink H. Vasculoprotective properties of the endothelial glycocalyx: Effects of fluid shear stress. Paper/Poster presented at: Journal of Internal Medicine; 2006;

47.	Vittum Z, Cocchiaro S, Mensah SA. Basal endothelial glycocalyx's response to shear stress: a review of structure, function, and clinical implications. *Front Cell Dev Biol*. 2024;12:1371769. doi: 10.3389/fcell.2024.1371769
