# Supplementary material for: Fluid shear stress-dependent modulation of the basal endothelial glycocalyx
Source: PLoS One. 2026 Jan 23;21(1):e0339318. doi: 10.1371/journal.pone.0339318 (PMC12829966; doi:10.1371/journal.pone.0339318)
Supplement: Attachment — Submitted filename: ResponseLetter_11_25.docx [file pone.0339318.s006.docx]

**Title:** Fluid shear stress-dependent modulation of the basal endothelial glycocalyx

**Journal:** PLOS One

**Submission ID:** PONE-D-25-37102

**Reviewer #1:**

**1.** Figures 2–5: The figure legends do not clearly indicate whether the cells were permeabilized. If this information is already included, it should be made explicit in the legends or methods section for clarity.

**Response to Reviewer**

We thank the reviewer for the helpful comment and apologize that our previous revision did not adequately improve the clarity. We have now updated each figure caption to clearly indicate the permeabilization status, ensuring that this information is explicit for every data block presented.

**Changes made to manuscript:**

Figure 2 through Figure 5 have been updated to say “apical and basal glycocalyx (GCX) expression in permeabilized human lung microvascular endothelial cells (HLMVECs)…” to clarify permeabilization status of the HLMVECs for each analysis.

***2*.** Figure 1: Statistical comparisons across time points are still missing. Given the observed decrease at 30 minutes and recovery at 12 hours, statistical analysis would strengthen the conclusions. In particular, for panels H and I, statistical analysis across time points appears feasible unless the authors consider it unnecessary due to the detailed data presented in Figures 2–4. If so, a brief clarification would be helpful.

**Response to Reviewer**

In Figure 1, as noted in our response to Comment 4.2 previously, the only comparisons presented are between the apical GCX signals of permeabilized and non-permeabilized HLMVECs under static culture, after 30 minutes, and after 12 hours of exposure to 10 dyn/cm² FSS. Accordingly, the three nonsignificant comparisons shown are the only analyses performed for Figure 1. To address your concern, we have expanded the muti-comparison ANOVA to include the temporal statistical comparisons. We have included these extra comparisons in an updated version of Figure 1, just for your review here. As you can see, the only significance added reflects trends in the permeabilized group, also shown in Figure 2. However, we believe that these comparisons do not add to the conclusions drawn from the figure. Rather, they may distract the reader from the main comparison and conclusion we are drawing from Figure 1 which is that the permeabilization treatment does not significantly affect apical GCX metrics. As permeabilization can affect membrane structure, potentially compromising the cell shape and structure or GCX itself, we feel that bringing only the comparisons that directly probe the effect of permeabilization between groups of the same shear exposure to the readers focus is most important here.

Also, as you pointed out, far more detailed comparisons on the temporal response are drawn and discussed in the latter figures. The temporal FSS responses of both apical and basal GCX metrics, as well as the comparisons between apical and basal measurements for each FSS condition, are presented in Figures 2–4. Figure 5 further summarizes the effects of different FSS rates and the temporal responses of apical and basal metrics separately. The figure legends and results sections have been revised for clarity.
